# CD6-Directed Immunotherapy Targets Breast Cancer Stem Cell Function and Enhances Immune-Mediated Cytotoxicity in Triple-Negative Breast Cancer

**DOI:** 10.3390/biom16081202

**Published:** 2026-08-17

**Authors:** Mikel Gurrea-Rubio, Sophie Sloan, Aditya Chada, Camila I. Amarista, Kohei Maeda, Phillip L. Campbell, Pei-Suen Tsou, Laura A. Cooney, Max S. Wicha, David A. Fox

**Affiliations:** 1 Department of Internal Medicine, Division of Rheumatology, University of Michigan, Ann Arbor, MI 48109, USA; 2 Department of Internal Medicine, Division of Hematology and Oncology, University of Michigan, Ann Arbor, MI 48109, USA

**Keywords:** triple-negative breast cancer, breast cancer stem-like cells, CD6, CD166, CD318, CD44

## Abstract

Triple-negative breast cancer (TNBC) is associated with recurrence, metastasis, and limited durable responses to immunotherapy, in part due to persistence of breast cancer stem cells (BCSCs). We investigated whether CD6-directed immunotherapy with the monoclonal antibody UMCD6 enhances immune-mediated killing and alters function of BCSC in stem cell-enriched TNBC models. The SUM-149 and SUM-159 cell lines were analyzed for CD6 ligand expression, cocultured with human peripheral blood mononuclear cells (PBMCs) treated with UMCD6, pembrolizumab, or isotype control, and assessed by live-cell cytotoxicity imaging, flow cytometry, soft agar colony formation, and extreme limiting dilution sphere assays. Both TNBC lines co-expressed the CD6 ligands CD44, CD166/ALCAM, and CD318/CDCP1. UMCD6 significantly increased PBMC-mediated apoptosis and reduced tumor cell survival in both models, with greater activity than pembrolizumab under these in vitro conditions. In surviving SUM-159 cells, UMCD6 reduced the ALDH^+^ population wit×hout significantly altering CD44^+^CD24^−^ frequency, indicating preferential effects on a distinct stem-like compartment. Functionally, UMCD6 decreased anchorage-independent colony formation and reduced sphere-forming frequency from 1/33.6 to 1/68.3 cells (*p* = 0.0186). These findings identify the CD6 ligand axis as a therapeutic vulnerability in BCSC-enriched TNBC and support further preclinical evaluation of CD6-directed immunotherapy as a strategy to enhance antitumor immunity while limiting tumor-initiating capacity.

## 1. Introduction

Breast cancer remains the most frequently diagnosed malignancy among women worldwide and a leading cause of cancer-related mortality [1]. Despite substantial advances in systemic therapy, recurrence and metastatic progression—particularly in aggressive subtypes such as triple-negative breast cancer (TNBC)—continue to limit long-term survival [2]. Increasing evidence suggests that disease relapse is driven by a subpopulation of tumor cells with stem-like properties, termed breast cancer stem cells (BCSCs), which possess enhanced clonogenicity, therapeutic resistance, immune evasion, and tumor-initiating capacity [3,4,5]. BCSCs are commonly enriched within ALDH1^+^ and CD44^+^CD24^−^ populations and are strongly associated with metastasis, treatment failure, and poor clinical outcomes [6]. Consequently, therapeutic strategies capable of simultaneously enhancing antitumor immunity and targeting stem-like tumor cells represent an important unmet clinical need.

CD6 is a lymphocyte-restricted surface glycoprotein expressed primarily on mature T cells and subsets of natural killer (NK) cells, where it functions as an important costimulatory receptor regulating lymphocyte activation, differentiation, and effector responses [7,8,9,10,11]. Through interactions with multiple ligands, CD6 contributes to immune synapse formation and has emerged as a therapeutic target in autoimmune disease and cancer [12]. In addition to interactions with galectin family members [13], the CD6 signaling axis is mediated through three principal cell-surface ligands: CD166 (activated leukocyte cell adhesion molecule [ALCAM]) [14], CD318 (CUB domain-containing protein 1 [CDCP1]) [15], and CD44 (H-CAM) [16].

Each of the three CD6 ligands has been implicated in aggressive breast cancer biology. CD44 is a canonical marker of breast cancer stem cells and plays a central role in tumor initiation, metastasis, and therapeutic resistance [17,18]. CD166 (ALCAM) is a cell adhesion molecule that regulates cell–cell interactions and tissue architecture and has been implicated in breast cancer progression; however, its prognostic significance remains controversial, with studies reporting both favorable and unfavorable clinical associations depending on tumor subtype, subcellular localization, and disease stage [19,20]. In contrast, CD318 is overexpressed in breast cancer and promotes invasion, metastatic dissemination, and disease progression [21,22]. The finding that stem cell-enriched TNBC populations express all three CD6 ligands raises the intriguing possibility that the CD6 signaling axis may represent a previously unrecognized therapeutic vulnerability within breast cancer stem cells.

Our previous studies demonstrated that targeting the CD6/CD318 axis with UMCD6, a monoclonal anti-CD6 antibody, enhances the cytotoxic activity of NK cells and CD8^+^ T cells against CD318-expressing solid tumors in vitro and in vivo [23,24]. However, whether CD6-directed immunotherapy influences breast cancer stem cell populations has not been investigated.

Here, we examined the expression of CD6 ligands in the stem cell-enriched TNBC cell lines SUM-149 and SUM-159 and evaluated whether CD6 targeting alters immune-mediated tumor cell killing and functional stemness. We demonstrate that these cell lines co-express the three main tumor-relevant cell-surface CD6 ligands examined here and that UMCD6 enhances peripheral blood mononuclear cell (PBMC)-mediated cytotoxicity while reducing ALDH activity, anchorage-independent growth, and sphere-forming capacity. Together, our findings identify the CD6 signaling axis as a potential therapeutic vulnerability within aggressive breast cancer stem-like populations and provide a rationale for further investigation of CD6-directed immunotherapy in TNBC.

## 2. Materials and Methods

### 2.1. Antibodies

The anti-human CD6 monoclonal antibody UMCD6 was generated and purified in our laboratory as previously described. Briefly, UMCD6 was affinity-purified from mouse ascites using Protein G affinity chromatography (Thermo Scientific, Wilmington, DE, USA) and desalted into phosphate-buffered saline prior to use. Pembrolizumab (anti-PD-1) was obtained from Merck (Darmstadt, Germany), and purified human IgG isotype control was purchased from Jackson ImmunoResearch (West Grove, PA, USA).

For flow cytometric analysis of CD6 ligands, fluorochrome-conjugated antibodies against CD166 (ALCAM; clone 3A6) and CD318 (CDCP1; clone CUB1) were purchased from BioLegend. Breast cancer stem cell populations were characterized using antibodies against CD44 (clone C44Mab-5) and CD24 (clone ML5) (BioLegend, San Diego, CA, USA).

### 2.2. Culture of Breast Cancer Stem Cell-Enriched Lines

Breast cancer stem cell-enriched triple-negative breast cancer (TNBC) cell lines SUM-159 and SUM-149 were obtained from the laboratory of Dr. Max Wicha (University of Michigan). SUM-159 cells were maintained in Ham’s F-12 medium supplemented with 5% heat-inactivated fetal bovine serum (FBS), 10 mM HEPES, insulin (5 μg/mL), and hydrocortisone (1 μg/mL). SUM-149 cells were maintained in Ham’s F-12 medium supplemented with 5% heat-inactivated FBS, epidermal growth factor (EGF; 20 ng/mL), insulin (5 μg/mL), and hydrocortisone (1 μg/mL). Cells were cultured at 37 °C in a humidified incubator with 5% CO_2_ and passaged using standard trypsinization procedures.

### 2.3. Immune-Mediated Cancer Cell Killing Assays

Real-time cytotoxicity assays were performed using the IncuCyte^®^ S3 Live-Cell Analysis System (version 2026A, Sartorius, Göttingen, Germany) as previously described [23,24]. Briefly, SUM-149 and SUM-159 cells were seeded into 96-well flat-bottom plates at 2 × 10^4^ cells per well and allowed to adhere overnight. Freshly isolated PBMCs were pre-incubated for 1 h at 37 °C with UMCD6, pembrolizumab, or human IgG isotype control (10 μg/mL each) before being added to tumor cells at an effector-to-target ratio of 2.5:1 (5 × 10^4^ PBMCs per 2 × 10^4^ cancer cells). UMCD6 was used at 10 µg/mL based on prior dose-ranging studies demonstrating that this concentration reproducibly induces CD6 modulation and enhances cytotoxic lymphocyte function without requiring higher antibody concentrations [23,24]. Caspase-3/7 Green Apoptosis Reagent (Sartorius) was added to each well at a final concentration of 5 μM to detect apoptotic tumor cells. Plates were placed into the IncuCyte system and imaged every 2 h for up to 5 days at 37 °C with 5% CO_2_. Four images were acquired per well using a 10X objective. Apoptotic tumor cells were quantified by caspase-3/7 green fluorescence, while tumor cell survival was determined by mKate2 nuclear fluorescence. A size-based object filter was applied to the green fluorescence channel to eliminate detection of PBMC-associated death events and nonspecific fluorescent artifacts. Images were analyzed using IncuCyte integrated software (version 2026A, Sartorius) with identical analysis parameters applied to all experimental groups. All conditions were performed in technical triplicate using PBMCs from 5–6 healthy donors.

### 2.4. Development of CD318-Knockdown MDA-MB-468 Cells

CD318 expression in MDA-MB-468 cells was knocked down using short hairpin RNA–encoding (shRNA-encoding) lentiviruses (Millipore Sigma, Burlington, MA, USA). Briefly, 5 × 10^4^ MDA-MB-468 cells were seeded in a 12-well plate overnight. The following day, cells were incubated with fresh media, 8 µg/mL hexadimethrine bromide and 5 × 10^5^ lentiviral particles encoding CD318/CDCP1-specific or nontarget shRNA (MOI = 2) for ~18 h. 48 h after transduction, cells were selected using 2 µg/mL puromycin in complete media for 2 weeks. Flow cytometric sorting based on CD318 expression was used to successfully select transduced cells. Wild-type and CD318-knockdown MDA-MB-468 cells were subsequently used as target cells in PBMC coculture assays performed under the same antibody treatment and IncuCyte imaging conditions described above, with tumor cell apoptosis quantified by nuclear caspase reporter fluorescence.

### 2.5. Flow Cytometry

Breast cancer cell lines were harvested using non-enzymatic dissociation buffer when appropriate to preserve surface antigens. After washing with Cell Staining Buffer (BioLegend, Cat# 420201), cells were incubated with Human TruStain FcX™ Fc receptor blocker (BioLegend, Cat# 422302) for 10 min at room temperature to prevent nonspecific binding. Cells were subsequently stained with Zombie Yellow™ Fixable Viability Dye (BioLegend) to exclude non-viable cells, followed by fluorochrome-conjugated antibodies against CD44, CD318 (CDCP1), CD166 (ALCAM), and CD24. Surface staining was performed for 30 min at 4 °C protected from light. Unstained controls and fluorescence minus one (FMO) controls were included for gating accuracy. Dead cells and debris were excluded based on forward scatter (FSC), side scatter (SSC), and high viability dye signal. Samples were acquired on a Cytek Aurora or Cytek Aurora Evo spectral flow cytometer (Cytek Biosciences, Fremont, CA, USA). At least 10,000 live events were acquired for each sample. Data were analyzed using FlowJo software (v10.10.0). Gating strategies are shown in Supplementary Figure S1. CD24 staining was used together with CD44 to identify the CD44^+^CD24^−^ breast cancer stem-like population.

### 2.6. ALDH Activity Assay

ALDH enzymatic activity was assessed using the ALDEFLUOR™ Kit (STEMCELL Technologies, Vancouver, BC, Canada, Cat# 01700) according to the manufacturer’s instructions. Briefly, tumor cells were incubated with the ALDH substrate (BAAA) in assay buffer for 30 min at 37 °C. A matched sample treated with the specific ALDH inhibitor diethylaminobenzaldehyde (DEAB) was used to define the negative gate. ALDH^+^ populations were quantified by flow cytometry after exclusion of dead cells and debris.

### 2.7. Isolation of Peripheral Blood Mononuclear Cells (PBMCs)

Peripheral blood was obtained from healthy adult donors after written consent and under institutional review board-approved protocols at the University of Michigan (Unique Surface Structures of Synovial Cells IRBMED 1988-0305; HUM00043197; approved date: 9 September 2025). Blood was collected into sodium heparin anticoagulant tubes and processed within 2 h of collection. PBMCs were isolated by density gradient centrifugation. Whole blood was diluted with PBS and layered over Ficoll-Paque solution (GE Healthcare, Chicago, IL, USA), followed by centrifugation at 400× *g* for 30 min at room temperature without brake. The mononuclear cell layer at the plasma-Ficoll interface was carefully collected, washed twice in PBS, and resuspended in RPMI-1640 medium supplemented with 10% FBS. Cell viability was assessed by trypan blue exclusion and routinely exceeded 95%. PBMCs were used immediately for cytotoxicity assays.

### 2.8. Recovery of Surviving Tumor Cells Following PBMC Coculture

To evaluate the impact of CD6 targeting on breast cancer stem cell phenotypes and clonogenicity, SUM-159 cells were cocultured with freshly isolated PBMCs pre-incubated with UMCD6 or human IgG isotype control (10 μg/mL each) for 5 days under the same conditions described for the IncuCyte cytotoxicity assays. At the end of the coculture period, non-adherent PBMCs together with detached apoptotic tumor cells were removed by gentle aspiration and repeated washing with phosphate-buffered saline (PBS). The remaining adherent tumor cells, representing the viable tumor cell population remaining after immune-mediated killing, were detached with trypsin-EDTA and collected for downstream analyses. Recovery of viable tumor cells and the absence of significant PBMC contamination were confirmed by flow cytometry demonstrating uniform CD318 expression. Recovered tumor cells were subsequently used for ALDEFLUOR analysis (ALDEFLUOR™ Kit, STEMCELL Technologies, Vancouver, BC, Canada, Cat # 01700), CD44/CD24 immunophenotyping, soft agar colony formation assays, and limiting dilution sphere-forming assays.

### 2.9. Soft Agar Colony Formation Assay

Anchorage-independent growth was assessed using a soft agar colony formation assay. A base layer of 0.6% agarose in complete culture medium was prepared in 6-well plates and allowed to solidify. Recovered viable SUM-159 tumor cells (1000 cells per well) were suspended in 0.35% agarose prepared in complete medium and layered over the base agar. After solidification, complete medium was added on top and replenished every 3 days. Cultures were maintained for 21 days at 37 °C with 5% CO_2_. At endpoint, colonies were stained with 0.005% crystal violet for visualization and imaged using brightfield microscopy. Macroscopic colonies (>50 μm diameter) were quantified using automated image analysis software (Quanty One).

### 2.10. Extreme Limiting Dilution Assay

Sphere-forming frequency was assessed using an extreme limiting dilution assay (ELDA). Following 5 days of coculture with PBMCs pretreated with UMCD6 (10 μg/mL) or human IgG isotype control (10 μg/mL), surviving adherent SUM-159 tumor cells were recovered as described above and plated into ultra-low attachment 96-well plates at limiting dilutions of 1, 10, 50, 100, 250, or 500 cells per well (12 replicate wells per dilution). Cells were cultured for 14 days in serum-free mammosphere medium consisting of DMEM/F12 supplemented with B-27 (1X), epidermal growth factor (EGF; 20 ng/mL), basic fibroblast growth factor (bFGF; 10 ng/mL), insulin (5 μg/mL), heparin (4 μg/mL), and hydrocortisone (1 μg/mL). Tumorspheres were defined as compact spherical colonies measuring ≥50 μm in diameter. Wells containing at least one tumorsphere were scored as positive. Cancer stem cell frequency was estimated using Extreme Limiting Dilution Analysis (ELDA) software [25].

### 2.11. Statistical Analysis

Statistical analyses were performed using GraphPad Prism (v10.4.1). Data are presented as mean ± standard deviation (SD) or mean ± standard error of the mean (SEM), as indicated in the figure legends. Comparisons between two groups were performed using unpaired two-tailed Student’s *t*-tests. Time-course cytotoxicity experiments were analyzed using two-way analysis of variance (ANOVA) with multiple-comparisons testing. Cancer stem cell frequencies were estimated using Extreme Limiting Dilution Analysis (ELDA), and differences between groups were evaluated using the ELDA likelihood ratio test. A two-sided *p* value < 0.05 was considered statistically significant.

## 3. Results

### 3.1. Breast Cancer Stem Cell-Enriched TNBC Cell Lines Express High Levels of CD6 Ligands

To determine whether breast cancer stem cell (BCSC)-enriched triple-negative breast cancer (TNBC) cell lines express ligands for CD6, we analyzed the surface expression of CD44, CD166/ALCAM, and CD318 by flow cytometry in SUM-159 and SUM-149 cells. These cell lines are well-established models enriched for stem-like breast cancer cells and have previously been reported to contain ALDH^+^ and CD44^+^CD24^−^ populations associated with tumor-initiating capacity and enhanced clonogenic potential. The flow cytometry gating strategy used for these analyses is shown in Supplementary Figure S1.

**Figure 1:**
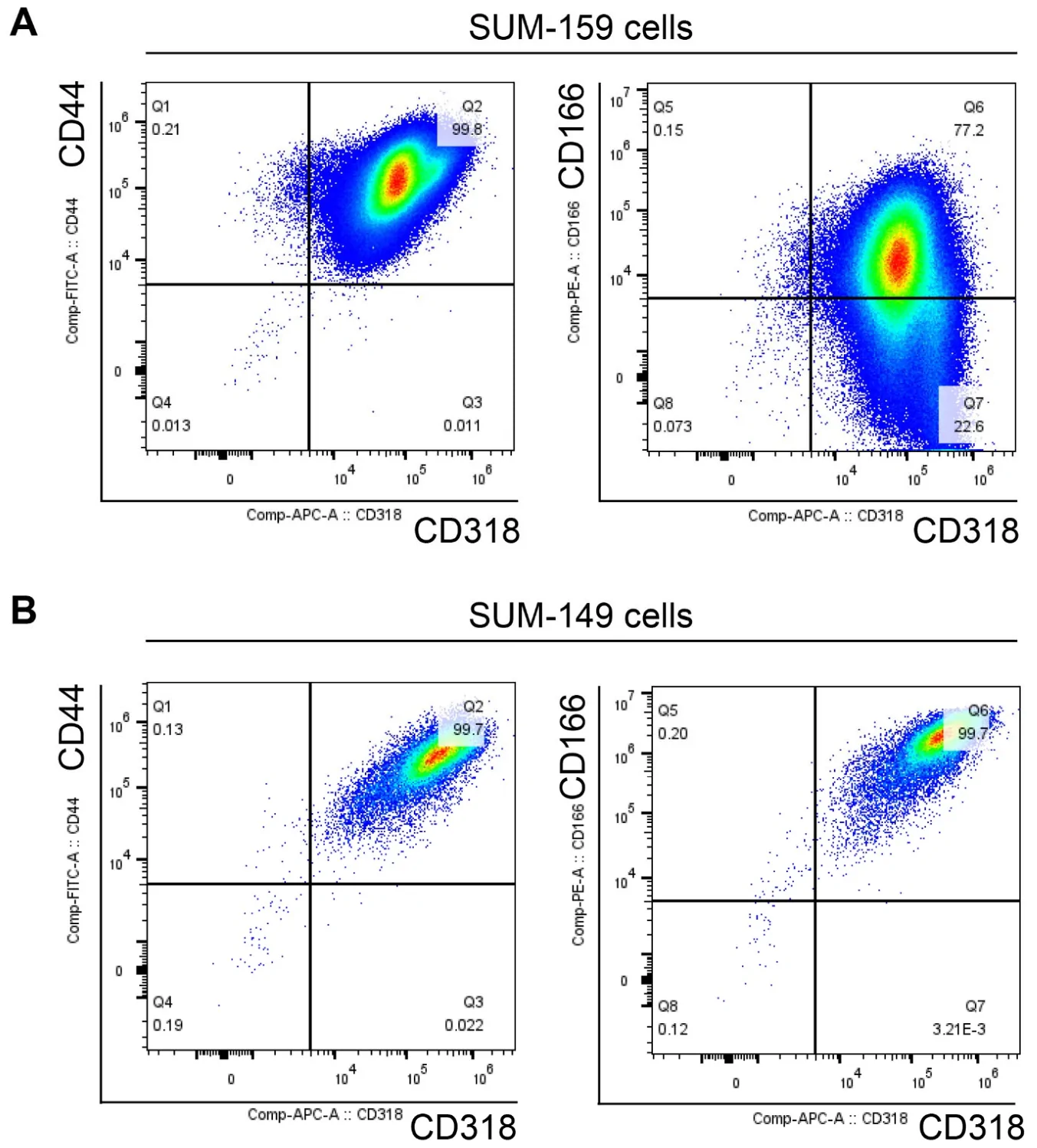
Surface expression of CD6 ligands in breast cancer stem cell-enriched cell lines. Representative flow cytometric analysis of the breast cancer stem cell-enriched triple-negative breast cancer cell lines SUM-159 (**A**) and SUM-149 (**B**). Representative biaxial density plots demonstrate the surface expression of CD44, CD166/ALCAM, and CD318. Left panels show co-expression of CD44 and CD318, while right panels show co-expression of CD166 and CD318. Both SUM-159 and SUM-149 cells exhibited high surface expression of CD318 together with CD44 and CD166. Percentages indicate the frequency of cells within each gated quadrant. Data shown are representative of independent experiments.

Flow cytometric analysis demonstrated robust expression of CD318 in both SUM-159 and SUM-149 cells (Figure 1). In SUM-159 cells, nearly all cells co-expressed CD44 and CD318, with approximately 99.8% of cells detected in the double-positive quadrant. In contrast, CD166/CD318 co-expression was less uniform: approximately 77.2% of SUM-159 cells were CD166^+^CD318^+^, whereas approximately 22.6% were CD166^−^CD318^+^. Thus, although SUM-159 cells broadly expressed multiple CD6 ligands, CD166 expression was more heterogeneous than CD44 or CD318 expression. SUM-149 cells displayed a more uniformly ligand-positive phenotype, with approximately 99.7% of cells co-expressing CD44 and CD318 and nearly all cells co-expressing CD166 and CD318. These findings demonstrate that BCSC-enriched TNBC cell lines broadly express all three known CD6 ligands. Although these ligands contribute distinct biological functions in breast cancer biology, their simultaneous expression within stem cell-enriched TNBC populations suggests that the CD6 signaling axis may represent a previously unrecognized therapeutic vulnerability.

### 3.2. UMCD6 Enhances PBMC-Mediated Killing of TNBC Cells

To determine whether CD6 targeting promotes immune-mediated killing of TNBC cells, SUM-159 cells were cocultured with human PBMCs pretreated with UMCD6, pembrolizumab, or isotype control IgG and monitored in real time using IncuCyte live-cell imaging. Apoptosis was quantified by caspase-3/7 activation, while tumor cell survival was assessed by longitudinal enumeration of viable SUM-159 cells.

**Figure 2:**
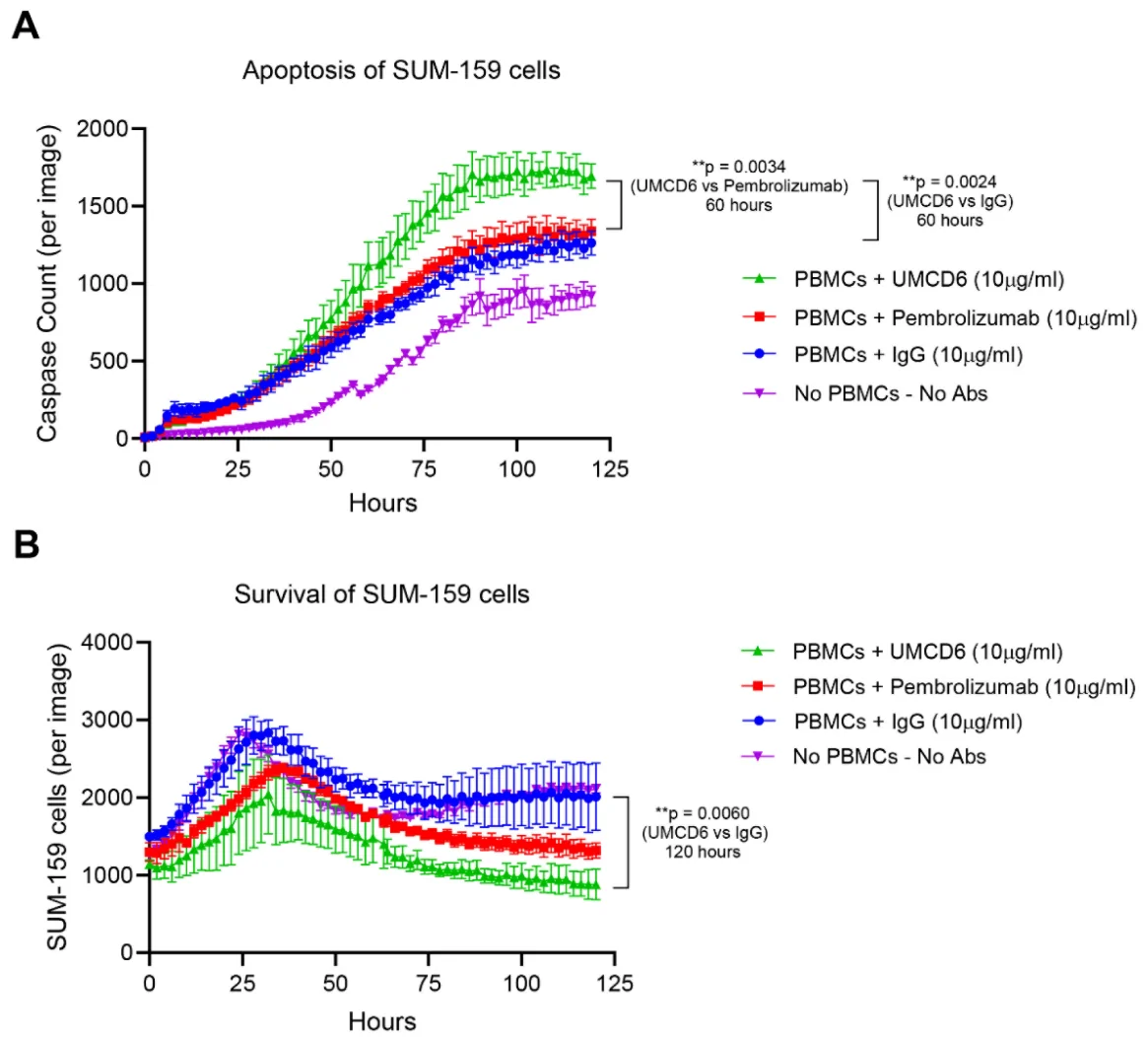

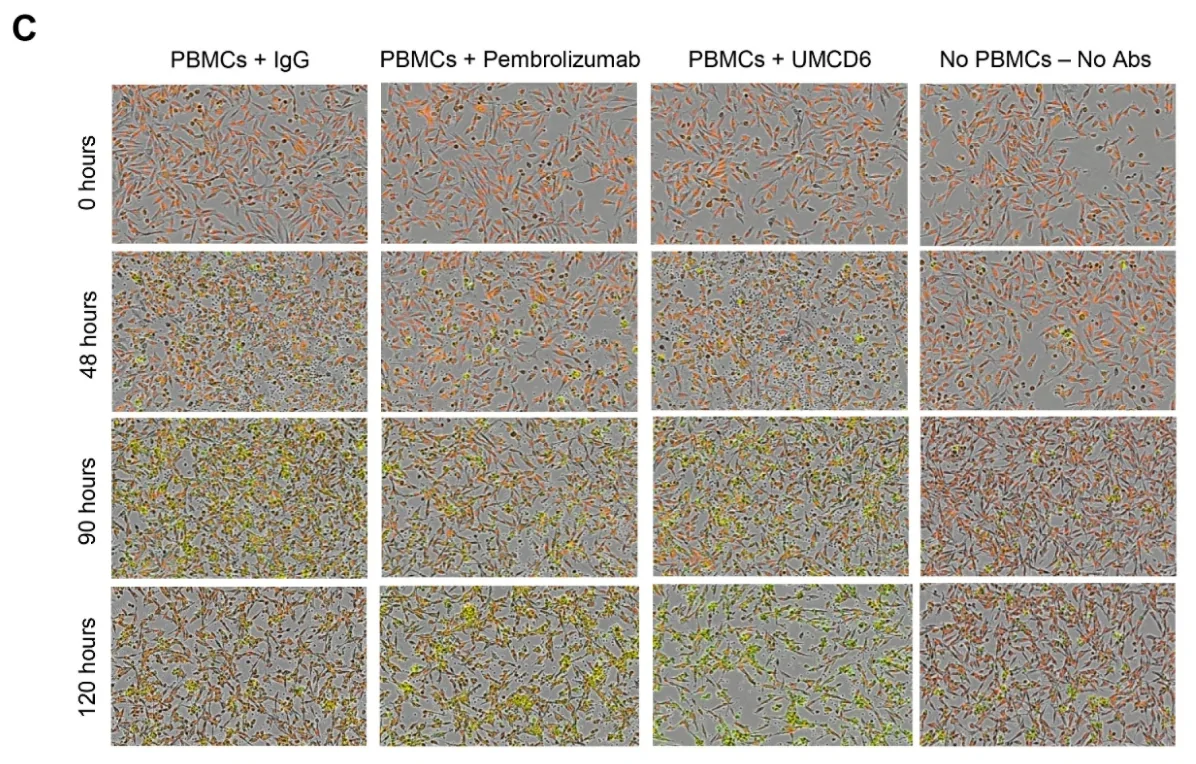
UMCD6 promotes immune-mediated killing of breast cancer stem cell-enriched SUM-159 TNBC cells. (**A**,**B**) SUM-159 triple-negative breast cancer cells were cocultured with human peripheral blood mononuclear cells (PBMCs) that had been pre-incubated for 1 h at 37 °C with UMCD6, anti-PD-1 (pembrolizumab), or IgG control (10 μg/mL). Tumor cell apoptosis was monitored in real time using a caspase-3/7 fluorescent reagent and quantified using the IncuCyte^®^ live-cell imaging system. Tumor cell survival was simultaneously assessed by measuring the number of viable SUM-159 cells over time. UMCD6 significantly increased tumor cell apoptosis compared with IgG-treated control (*p* = 0.025) and pembrolizumab (*p* = 0.0034) beginning approximately 60 h after initiation of coculture and persisting throughout the remainder of the experiment. UMCD6 also significantly reduced tumor cell survival compared with IgG-treated control (*p* = 0.006). Statistical significance was determined by two-way ANOVA with multiple-comparisons testing. Data are presented as mean ± SEM. (**C**) Representative IncuCyte^®^ images of SUM-159 cells cultured with PBMCs and treated with IgG control, anti-PD-1 (pembrolizumab), or UMCD6, together with a tumor-cell-only control lacking PBMCs and antibodies. Images were acquired at 0, 48, 90, and 120 h. Red fluorescence indicates viable tumor cells, whereas green fluorescence indicates caspase-3/7 activation and apoptotic cell death. Cocultures treated with UMCD6 exhibited increased caspase activity and decreased tumor cell density over time compared with anti-PD-1- and IgG-treated controls. All images were acquired at 10X magnification.

UMCD6 treatment significantly increased tumor cell apoptosis compared with IgG-treated cocultures and induced greater apoptotic activity than pembrolizumab-treated controls (Figure 2A). Enhanced caspase-3/7 activation became evident approximately 60 h after initiation of coculture and continued throughout the remainder of the experiment. Consistent with these findings, UMCD6 significantly reduced the number of surviving SUM-159 cells compared with IgG-treated controls and demonstrated greater activity relative to anti-PD-1 treatment (Figure 2B). Representative IncuCyte images confirmed progressive tumor cell elimination and increased apoptotic cell death in UMCD6-treated cultures (Figure 2C).

To determine whether this activity was restricted to a single TNBC model, identical experiments were performed using the independent BCSC-enriched TNBC cell line SUM-149. Similar to SUM-159 cells, UMCD6 significantly increased apoptosis and reduced tumor cell survival compared with IgG-treated controls and exhibited greater antitumor activity than pembrolizumab (Supplementary Figure S2). Together, these findings demonstrate that UMCD6 enhances PBMC-mediated cytotoxicity against multiple TNBC cell lines enriched for breast cancer stem cell populations.

To assess whether CD318 expression contributes to UMCD6-enhanced immune-mediated tumor cell killing, we performed additional PBMC coculture assays using wild-type and CD318-knockdown MDA-MB-468 breast cancer cells. Target cell apoptosis was monitored using IncuCyte live-cell imaging by quantifying nuclear caspase reporter fluorescence. UMCD6 enhanced PBMC-mediated apoptosis of CD318-expressing MDA-MB-468 cells, whereas this effect was reduced in CD318-knockdown cells (Supplementary Figure S3). These findings provide supportive evidence that tumor-cell CD318 expression contributes to UMCD6-enhanced immune-mediated apoptosis, although they do not exclude contributions from other CD6 ligands or tumor-intrinsic effects of CD318 modulation.

### 3.3. UMCD6 Reduces the ALDH^+^ Breast Cancer Stem Cell Population

**Figure 3:**
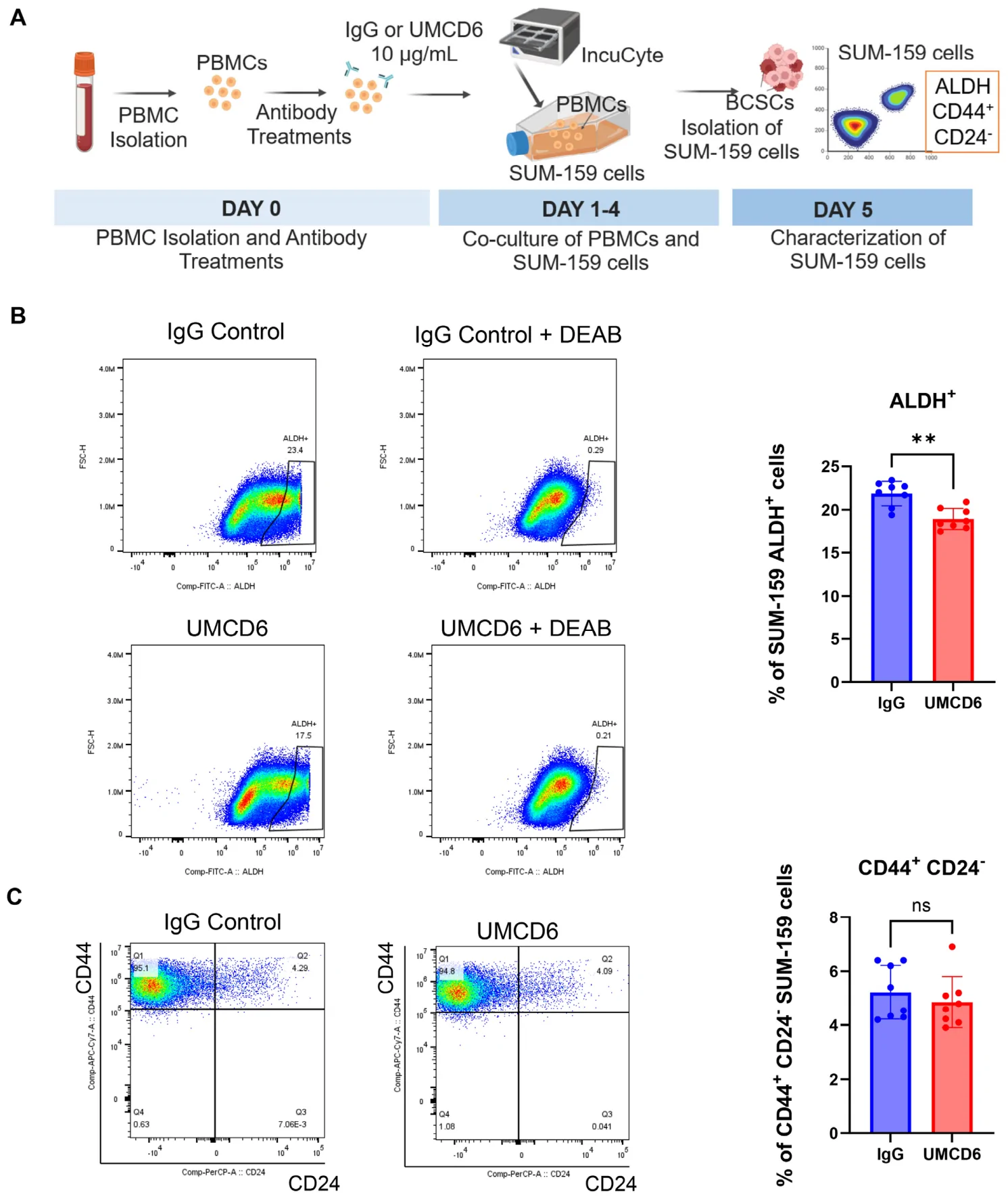
UMCD6 reduces the ALDH^+^ breast cancer stem cell population in SUM-159 cells following coculture with PBMCs. (**A**) Experimental schematic. Human PBMCs were isolated and treated with UMCD6 or isotype control IgG (10 μg/mL) prior to coculture with SUM-159 breast cancer cells. After 5 days of coculture, surviving SUM-159 cells were recovered and analyzed by flow cytometry for breast cancer stem cell-associated markers, including ALDH activity and CD44/CD24 expression. (**B**) ALDH activity was assessed using the ALDEFLUOR assay in surviving SUM-159 cells. Representative flow cytometry plots from IgG- and UMCD6-treated cocultures are shown with corresponding DEAB controls. Quantification demonstrated a significant reduction in the percentage of ALDH^+^ SUM-159 cells following UMCD6 treatment compared with IgG control, indicating a reduction in the ALDH^+^ tumor cell population. Each symbol represents an independent experiment. Bars represent mean ± SD. *p* < 0.01, unpaired two-tailed *t*-test. (**C**) The frequency of CD44^+^CD24^−^ cells was evaluated by flow cytometry in surviving SUM-159 cells. Representative dot plots and quantification are shown. No significant differences in CD44^+^CD24^−^ frequency were observed between treatment groups. Each symbol represents an independent experiment. Bars represent mean ± SD. ns, not significant by unpaired two-tailed *t*-test. “**” indicates *p* < 0.01.

Because breast cancer stem cells contribute to tumor initiation, therapeutic resistance, and disease recurrence, we next examined whether UMCD6 selectively affected stem-like tumor cell populations that survived PBMC-mediated immune challenge. Human PBMCs were treated with UMCD6 or isotype control IgG and cocultured with SUM-159 cells for 5 days. Surviving tumor cells were then isolated and analyzed for established breast cancer stem cell markers (Figure 3A).

Assessment of aldehyde dehydrogenase activity using the ALDEFLUOR assay revealed a significant reduction in the frequency of ALDH^+^ SUM-159 cells following UMCD6 treatment (Figure 3B). Representative flow cytometric plots demonstrated a decrease in the ALDH-positive population in UMCD6-treated cultures relative to IgG-treated controls, and quantification across independent experiments confirmed a significant reduction in ALDH^+^ cells. These findings indicate that UMCD6-mediated immune killing preferentially reduces a tumor cell population characterized by high ALDH activity, a functional marker associated with breast cancer stem cells.

In contrast, analysis of CD44 and CD24 expression demonstrated no significant difference in the frequency of CD44^+^CD24^−^ cells between UMCD6- and IgG-treated cultures (Figure 3C). Thus, while UMCD6 reduced the ALDH^+^ compartment, it did not substantially alter the overall frequency of cells expressing the classical CD44^+^CD24^−^ phenotype. These observations suggest that ALDH activity may identify a functionally distinct tumor-initiating population preferentially susceptible to UMCD6-mediated immune elimination.

### 3.4. UMCD6 Impairs Clonogenic Growth of Surviving TNBC Cells

To determine whether UMCD6-induced depletion of ALDH^+^ cells translated into functional impairment of tumor cell self-renewal, surviving SUM-159 cells recovered from PBMC cocultures were assessed in a soft agar colony formation assay. Cells were replated in semisolid medium and cultured for 21 days to assess anchorage-independent growth, a functional measure of clonogenic potential.

**Figure 4:**
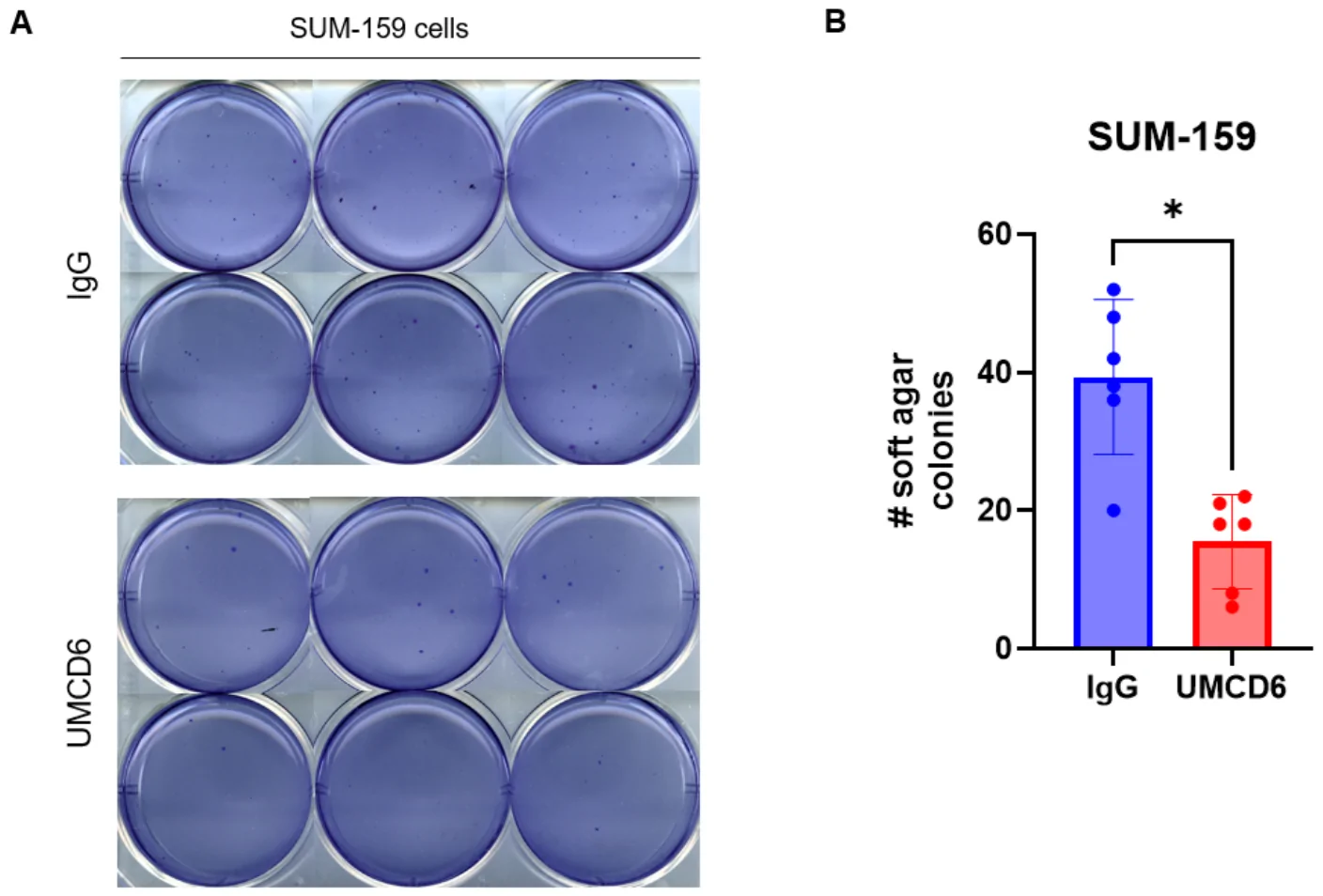
UMCD6 impairs anchorage-independent growth of surviving SUM-159 cells after PBMC-mediated immune challenge. (**A**) SUM-159 cells were cocultured with human PBMCs in the presence of UMCD6 or isotype control IgG for 5 days. Surviving tumor cells were recovered and replated in soft agar to assess clonogenic growth. Representative crystal violet-stained cultures after 21 days are shown. (**B**) Quantification of colonies demonstrated a significant reduction in colony formation by SUM-159 cells previously exposed to UMCD6. Bars represent mean ± SD, with each dot representing one replicate well from a representative experiment containing six wells per condition. The experiment was repeated three independent times with similar results. Statistical significance was determined by unpaired two-tailed Student’s *t*-test; *p* = 0.0169. “*” indicates *p* < 0.05.

Representative crystal violet-stained cultures demonstrated a visible reduction in colony formation among cells previously exposed to UMCD6 compared with IgG-treated controls (Figure 4A). Quantitative analysis confirmed a significant decrease in the number of colonies formed by UMCD6-treated cells (Figure 4B). These findings indicate that UMCD6-mediated immune challenge not only reduces ALDH^+^ tumor-initiating cells but also impairs the capacity of surviving tumor cells to undergo anchorage-independent growth.

### 3.5. UMCD6 Reduces Sphere-Forming Frequency of Breast Cancer Stem-like Cells

**Figure 5:**
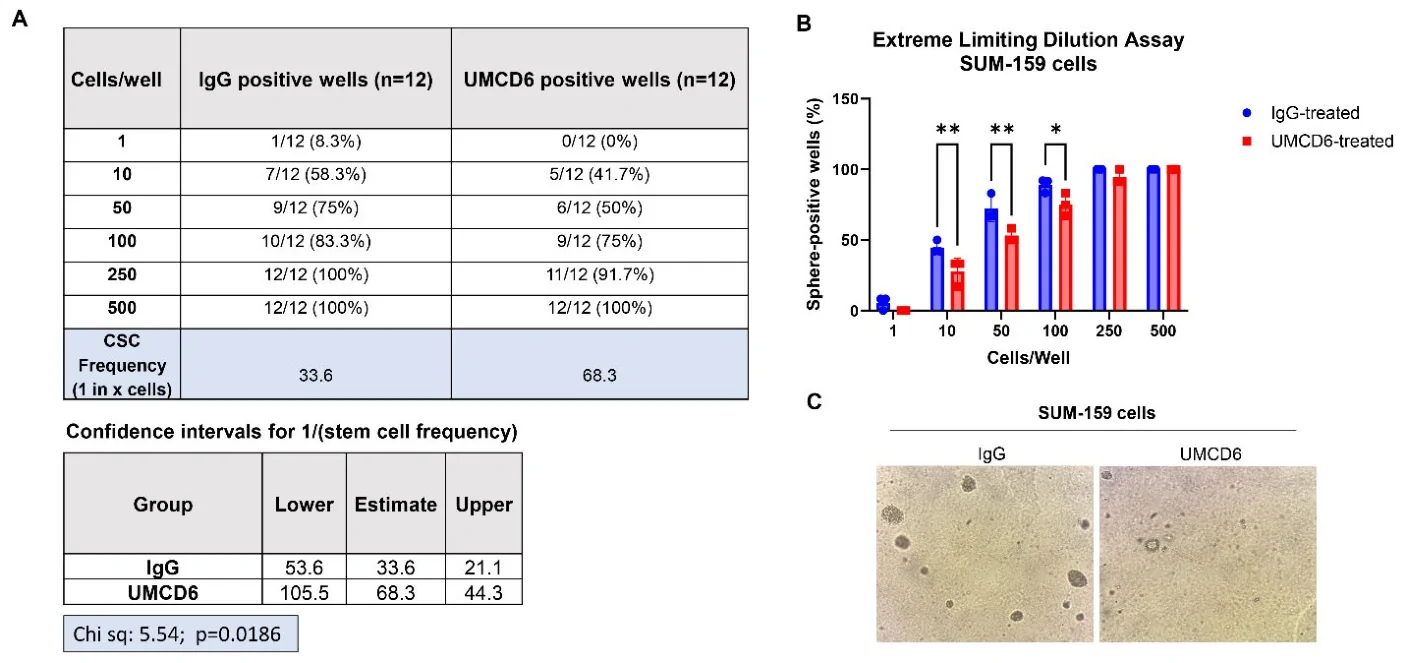
UMCD6 reduces the frequency of sphere-forming SUM-159 breast cancer cells following coculture with PBMCs. (**A**) Surviving SUM-159 cells were recovered after 5 days of coculture with human PBMCs in the presence of UMCD6 or isotype control IgG and subjected to an extreme limiting dilution assay (ELDA). Cells were plated at the indicated densities (1–500 cells/well) in 96-well plates (*n* = 12 wells per dilution). After 14 days, wells containing at least one tumorsphere were scored as sphere-positive. The table summarizes the number and percentage of sphere-positive wells at each cell density from a representative experiment. Cancer stem cell (CSC) frequency was estimated by ELDA with corresponding 95% confidence intervals and likelihood ratio testing. UMCD6 significantly reduced the estimated CSC frequency compared with IgG-treated controls, from 1 in 33.6 cells to 1 in 68.3 cells (*p* = 0.0186). (**B**) Quantification of sphere-positive wells at each plating density. Data represent the mean ± SEM from three independent experiments performed with different PBMC donors. Statistical significance was determined by two-way ANOVA (*p* < 0.05, *p* < 0.01). (**C**) Representative brightfield images of tumorspheres generated from surviving SUM-159 cells after 14 days under tumorsphere culture conditions. Cells previously exposed to UMCD6 formed fewer and smaller tumorspheres than IgG-treated controls. Images are shown as representative examples of sphere formation. “*” indicates *p* < 0.05. “**” indicates *p* < 0.01.

To directly evaluate the effect of UMCD6 on tumor-initiating and self-renewing cell populations, surviving SUM-159 cells were analyzed using an extreme limiting dilution assay (ELDA). Following coculture with PBMCs and UMCD6 or IgG control, tumor cells were plated at limiting densities ranging from 1 to 500 cells per well and cultured under sphere-forming conditions for 14 days (Figure 5A).

UMCD6-treated cells exhibited a reduced frequency of sphere-positive wells across multiple cell dilutions compared with IgG-treated controls. ELDA demonstrated a significant reduction in sphere-forming cell frequency following UMCD6 exposure, with estimated cancer stem cell frequencies decreasing from 1 in 33.6 cells in the IgG-treated group to 1 in 68.3 cells in the UMCD6-treated group (Figure 5A). This corresponded to an approximately 2-fold reduction in functional cancer stem cell frequency and was significant by likelihood ratio analysis (*p* = 0.0186). Representative images obtained at the endpoint of the assay demonstrated fewer and less frequent spheres in UMCD6-treated cultures compared with controls (Figure 5B). Together with the reductions in ALDH^+^ cells and anchorage-independent colony formation, these findings provide complementary phenotypic and functional evidence that UMCD6 depletes tumor cell populations with stem-like and self-renewing properties. Collectively, these results demonstrate that CD6-directed immunotherapy not only enhances immune-mediated killing of TNBC cells but also diminishes functional properties associated with breast cancer stem cells.

## 4. Discussion

Breast cancer stem cells (BCSCs) contribute to tumor initiation, therapeutic resistance, metastatic dissemination, and disease recurrence [17,26]. Although immune checkpoint inhibitors have improved outcomes for subsets of patients with triple-negative breast cancer (TNBC), durable responses remain limited, in part because stem-like tumor cell populations frequently evade immune-mediated elimination [27,28,29]. In the present study, we demonstrate that the BCSC-enriched TNBC cell lines SUM-149 and SUM-159 co-express the three principal tumor-relevant cell-surface CD6 ligands examined here—CD44, CD166, and CD318—and that targeting CD6 with the monoclonal antibody UMCD6 enhances PBMC-mediated cytotoxicity while reducing multiple phenotypic and functional properties associated with breast cancer stem cells, including ALDH activity, anchorage-independent growth, and sphere-forming frequency. Collectively, these findings identify the CD6 signaling axis as a previously unrecognized therapeutic vulnerability within stem cell-enriched TNBC populations.

One of the most intriguing observations from this study is the coordinated expression of CD44, CD166/ALCAM, and CD318/CDCP1 by stem cell-enriched TNBC cells. Rather than serving identical biological functions, these ligands contribute complementary aspects of aggressive tumor biology. CD44 is a canonical marker of breast cancer stem cells and is closely linked to tumor initiation, therapeutic resistance, and metastatic progression [6,17]. CD318 promotes invasion, metastatic dissemination, and poor clinical outcome [21,22,30], whereas CD166 (ALCAM) functions primarily as a cell adhesion molecule regulating cell–cell interactions within the tumor microenvironment, although its prognostic significance in breast cancer remains context-dependent [19,31]. The lower frequency of CD166/CD318 double-positive cells in SUM-159 compared with SUM-149 suggests heterogeneity in ALCAM/CD166 expression among stem cell-enriched TNBC models, consistent with prior reports that the biological and prognostic significance of CD166 in breast cancer is context-dependent. The simultaneous expression of these complementary ligands raises the possibility that the CD6 ligand axis may intersect with multiple biological processes relevant to breast cancer progression, including stemness, adhesion, immune interactions, and metastatic potential.

A major finding of this study is that UMCD6 enhanced PBMC-mediated killing of TNBC cells. In both SUM-149 and SUM-159 models, UMCD6 increased caspase-3/7 activation and significantly reduced tumor cell survival compared with isotype-treated controls. UMCD6 also showed greater activity than pembrolizumab under the defined in vitro PBMC coculture conditions tested. This comparison should be interpreted cautiously. Pembrolizumab acts primarily by relieving PD-1-mediated inhibition of antigen-experienced or exhausted T cells, and its activity depends on tumor antigen recognition, PD-1/PD-L1 interactions, and the immune activation state of the effector cells. These features may not be fully reproduced in short-term cocultures using healthy-donor PBMCs. Therefore, the current data demonstrate differential activity under the defined in vitro conditions tested but should not be interpreted as evidence of clinical superiority of UMCD6 over PD-1 blockade. These findings are consistent with previous studies demonstrating that CD6 targeting promotes activation of cytotoxic lymphocytes, including NK cells and CD8^+^ T cells, thereby enhancing antitumor immunity [23,24]. Because CD6 is expressed by both T cells and subsets of NK cells, UMCD6 may simultaneously augment multiple cytotoxic effector populations. This mechanism differs fundamentally from conventional PD-1 blockade, which primarily relieves inhibitory signaling within pre-existing T-cell responses [32].

Because CD6 is primarily expressed on lymphocytes, UMCD6 is expected to act on immune effector cells rather than directly on TNBC cells. Consistent with this interpretation, SUM-149 and SUM-159 cells lacked detectable CD6 expression by flow cytometry, whereas CD6 was readily detected on PBMC populations. Tumor-cell-only controls showed continued tumor cell growth in the absence of PBMCs, supporting the requirement for immune effector cells in this assay. Future studies including antibody-alone conditions across additional TNBC models would further exclude direct tumor-cell effects.

Importantly, the effects of UMCD6 extended beyond bulk tumor cell killing and were associated with reductions in stem cell-associated phenotypes. Following coculture with PBMCs, UMCD6 significantly reduced the frequency of ALDH^+^ tumor cells, a population strongly associated with tumor-initiating capacity and poor clinical outcome in breast cancer. In contrast, the proportion of CD44^+^CD24^−^ cells remained largely unchanged. Although initially unexpected, this observation is consistent with growing evidence that ALDH activity and CD44^+^CD24^−^ expression identify overlapping but biologically distinct breast cancer stem cell populations. Previous studies have shown that ALDH^+^ cells frequently exhibit greater proliferative and tumor-initiating potential, whereas CD44^+^CD24^−^ cells may represent a more mesenchymal and invasive stem-like state [33,34]. Our findings therefore suggest that UMCD6 preferentially affects a functionally relevant ALDH-defined tumor-initiating population rather than broadly altering expression of canonical stem cell surface markers.

The functional consequences of this reduction in stem-like cells were further demonstrated using complementary assays of clonogenicity and self-renewal. Tumor cells surviving UMCD6-mediated immune challenge displayed significantly reduced anchorage-independent growth in soft agar, indicating impaired clonogenic potential. Moreover, extreme limiting dilution analysis demonstrated an approximately two-fold reduction in functional cancer stem cell frequency, with sphere-forming frequency decreasing from one cancer stem cell in 33.6 cells to one in 68.3 cells following UMCD6 treatment. Importantly, these assays evaluate distinct aspects of breast cancer stem cell biology. ALDH activity identifies a phenotypic stem cell population [33], whereas soft agar colony formation and ELDA assess functional properties associated with clonogenicity, self-renewal, and tumor-initiating capacity [25]. The concordance of these independent assays therefore provides complementary phenotypic and functional evidence that UMCD6 influences breast cancer stem-like cell behavior rather than simply altering expression of stem cell-associated markers.

The mechanisms responsible for the preferential reduction in functional breast cancer stem-like cells remain to be fully elucidated. One possibility is that BCSCs are intrinsically more susceptible to enhanced NK-cell and CD8+ T-cell cytotoxicity induced by CD6 targeting. Alternatively, ligand-rich tumor cells may form distinct immune synapse interactions with CD6-expressing lymphocytes that are altered by UMCD6 treatment. UMCD6 is proposed to act primarily on CD6-expressing lymphocytes rather than by directly increasing natural CD6-ligand binding on tumor cells. The expression of CD44, CD166/ALCAM, and CD318/CDCP1 by TNBC cells may contribute to a CD6 ligand-rich tumor-immune interface, but the present data do not demonstrate that UMCD6 enhances natural ligand engagement. Instead, UMCD6 may promote cytotoxic lymphocyte function through antibody-induced CD6 clustering, agonistic signaling, or modulation of CD6-dependent immune synapse biology. Supportive CD318-knockdown data suggest that CD318 expression contributes to UMCD6-enhanced immune-mediated apoptosis, but complete CD6-ligand knockout experiments may be difficult to interpret because CD44, CD166, and CD318 each regulate tumor cell adhesion, survival, invasion, apoptotic sensitivity, and stem-like properties independently of CD6 binding. Future studies using epitope mapping, ligand-competition assays, inducible knockdown, controlled re-expression, and ligand-specific rescue systems will be needed to define the relative contribution of each CD6 ligand.

Interestingly, a recent transcriptomic analysis of patients with TNBC led by Li et al. has identified CD6 as a compelling prognostic immune gene associated with increased CD8^+^ T-cell infiltration and a more favorable predicted response to immunotherapy, further supporting the potential clinical relevance of the CD6 axis in TNBC [35]. Although that study was observational and did not investigate CD6-directed therapy, its findings complement our experimental data by independently implicating CD6 in productive antitumor immunity.

Several limitations should be considered. First, these studies were performed using in vitro PBMC coculture systems and functional stem cell assays rather than in vivo TNBC models. Because UMCD6 targets human CD6-expressing lymphocytes, conventional immunodeficient xenograft systems may not adequately model the relevant human immune compartment, and future studies using humanized immune systems, syngeneic surrogate approaches, or patient-derived tumor models will be required. Second, although UMCD6 enhanced PBMC-mediated cytotoxicity, the specific immune effector populations responsible for eliminating stem-like tumor cells were not defined. Prior studies suggest potential contributions from both NK cells and CD8+ T cells, but subset-depletion and purified effector cell studies will be needed. Third, although the current study demonstrates co-expression of CD44, CD166, and CD318 and provides supportive CD318-knockdown data, the relative contribution of each CD6 ligand remains unresolved. Complete CD6-ligand knockout experiments may be difficult to interpret because CD44, CD166, and CD318 regulate tumor-intrinsic processes, including adhesion, survival, invasion, apoptotic sensitivity, and stem-like properties. Fourth, the structural relationship between UMCD6 binding and natural CD6-ligand engagement was not defined; future epitope mapping and ligand-competition studies will be required. Fifth, pembrolizumab was included as an in vitro comparator in the cytotoxicity assays but was not included in the downstream ALDH, soft agar, or ELDAs. Therefore, the functional stemness data demonstrate effects of UMCD6 relative to isotype control and should not be interpreted as demonstrating superiority over PD-1 blockade in depletion of breast cancer stem-like cells. Future studies comparing CD6-directed therapy with checkpoint blockade, alone and in combination, in antigen-experienced or humanized immune systems will be needed. Finally, although immune-mediated cytotoxicity was confirmed in both SUM-149 and SUM-159 cells, downstream ALDH, soft agar, and ELDA analyses were performed primarily in SUM-159 cells, and additional TNBC models will be needed to establish generalizability.

Overall, these findings support a model in which CD6-directed immunotherapy may simultaneously enhance antitumor immune effector function and reduce the functional capacity of tumor cell populations associated with breast cancer stemness. The observation that UMCD6 affected ALDH activity, clonogenic growth, and sphere-forming capacity suggests that its effects extend beyond bulk tumor cell elimination and may influence properties relevant to tumor persistence and recurrence. However, the present findings remain preclinical and were obtained primarily in in vitro PBMC coculture systems, underscoring the need to determine whether these effects are maintained in more physiologically relevant immune and tumor microenvironments. Future studies using humanized immune systems, patient-derived models, and combination approaches with immune checkpoint blockade will be important to establish the therapeutic potential of CD6-directed strategies in aggressive TNBC.

## 5. Conclusions

This study identifies the CD6 ligand axis as a potential therapeutic vulnerability in breast cancer stem cell-enriched TNBC. CD6-directed immunotherapy with UMCD6 enhanced PBMC-mediated tumor cell killing and reduced functional properties associated with breast cancer stem-like cells, including ALDH activity, anchorage-independent growth, and sphere-forming capacity. Supportive CD318-knockdown experiments further implicate tumor-cell CD318 in UMCD6-enhanced immune-mediated apoptosis, although the contribution of individual CD6 ligands remains to be defined. These findings provide proof of concept for CD6-directed immunotherapy as a strategy to target both tumor cell survival and stem-like properties. Further validation in humanized immune systems, in vivo models, and patient-derived tumors will be necessary to determine the translational potential of this approach, including its possible use in combination with immune checkpoint blockade.

## Data Availability

The original contributions presented in this study are included in the article/Supplementary Material. Further inquiries can be directed to the corresponding author.

## Supplementary Materials

The following supporting information can be downloaded at: https://www.mdpi.com/article/10.3390/biom16081202/s1, Figure S1: Gating strategy and surface expression of CD6 ligands in breast cancer stem cell lines; Figure S2: UMCD6 enhances PBMC-mediated killing of SUM-149 breast cancer cells; Figure S3: CD318 knockdown reduces UMCD6-enhanced PBMC-mediated apoptosis of MDA-MB-468 breast cancer cells.
