# Supplementary material for: CD6-Directed Immunotherapy Targets Breast Cancer Stem Cell Function and Enhances Immune-Mediated Cytotoxicity in Triple-Negative Breast Cancer
Source: Biomolecules. 2026 Aug 17;16(8):1202. doi: 10.3390/biom16081202 (PMC13511485; doi:10.3390/biom16081202)
Supplement: Supplementary file 1 [file biomolecules-16-01202-s001.zip › biomolecules-4443198-supplementary.pdf]

## Supplementary Figure S1

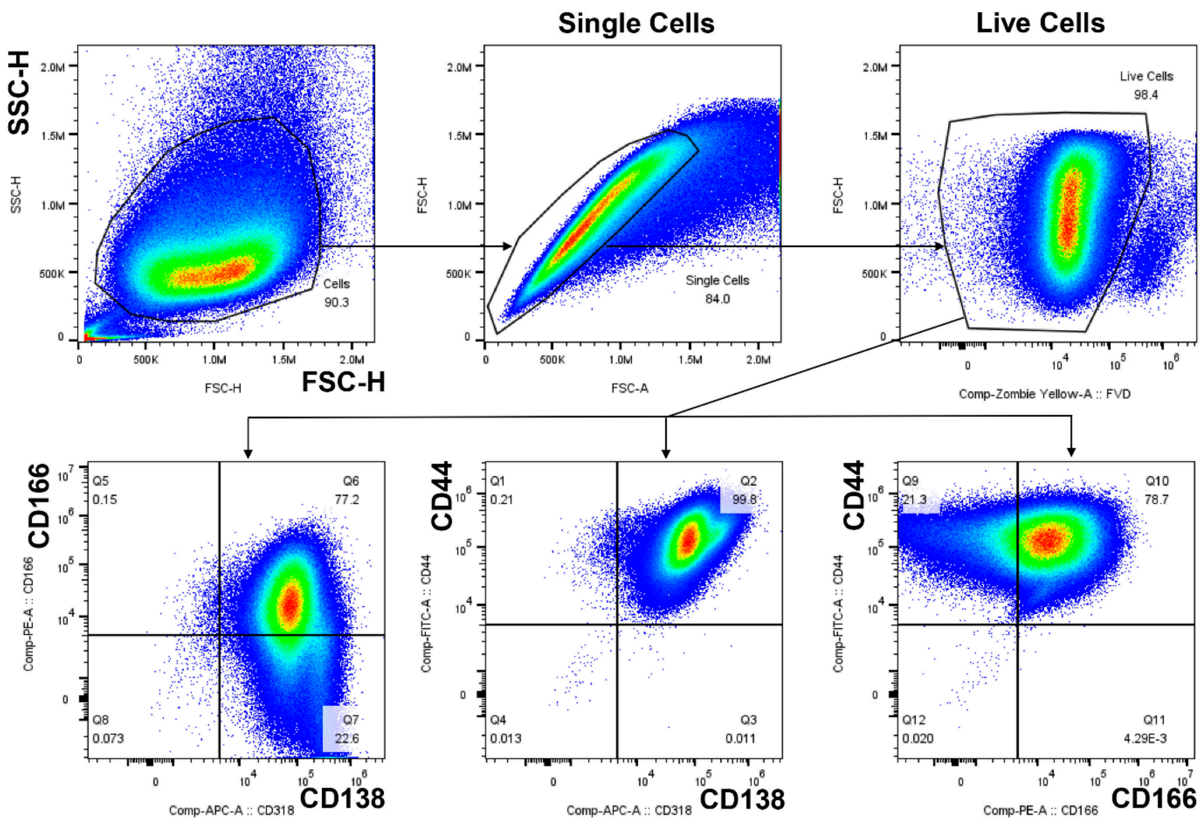


**Figure S1.** Gating strategy and surface expression of CD6 ligands in breast cancer stem cell lines. Representative flow cytometry gating strategy used to identify live single cells and assess expression of the CD6 ligands CD166 (ALCAM), CD318, and CD44 in breast cancer stem cell lines. Cells were first gated based on FSC-H versus SSC-H to exclude debris, followed by singlet discrimination using FSC-A versus FSC-H. Live cells were then identified by exclusion of the fixable viability dye (FVD). Representative biaxial plots demonstrate the distribution and relative frequency of CD166, CD318, and CD44 positive populations within the live cell gate.

## Supplementary Figure S2

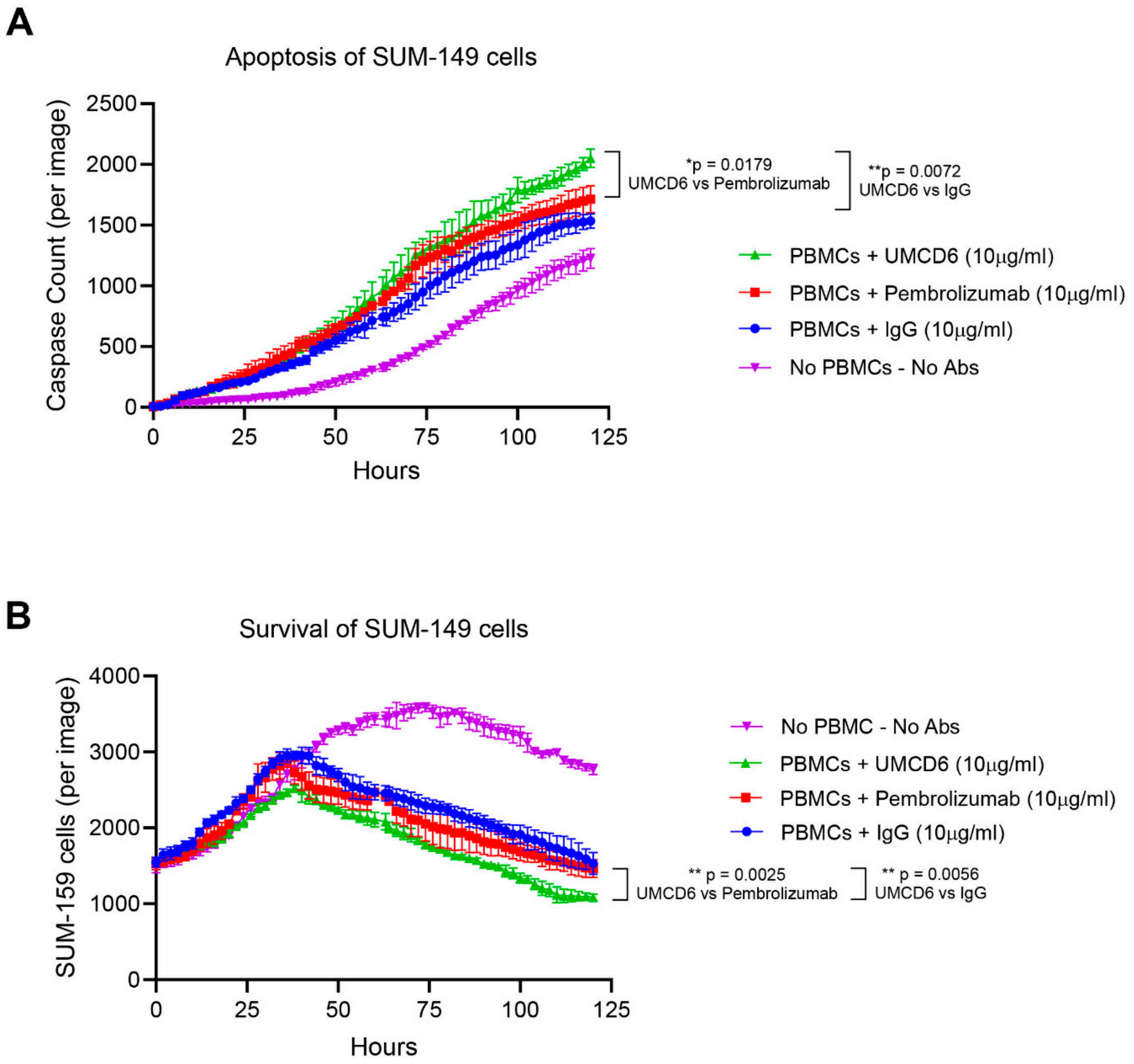


**Figure S2. UMCD6 enhances PBMC-mediated killing of SUM-149 breast cancer cells. (A)** Apoptosis of SUM-149 cells was monitored in real time using IncuCyte live-cell imaging. SUM-149 cells were cultured alone or cocultured with human PBMCs in the presence of UMCD6, pembrolizumab, or isotype control IgG (10 µg/mL each). Apoptotic cells were quantified by caspase-3/7 activity over time. UMCD6 significantly increased apoptosis of SUM-149 cells compared with IgG-treated controls *($P = 0.0072$)* and pembrolizumab-treated cocultures *($P = 0.0179$)*. **(B)** SUM-149 cell survival was quantified by IncuCyte live-cell imaging over the same time course. Coculture with PBMCs and UMCD6 significantly reduced the number of surviving SUM-149 cells compared with IgG-treated controls *($P = 0.0056$)* and pembrolizumab-treated cocultures *($P = 0.0035$)*. SUM-149 cells cultured in the absence of PBMCs showed continued expansion throughout the experiment. Data are presented as mean ± SEM. Statistical significance was determined by two-way ANOVA with multiple-comparisons testing.

**Supplementary Figure S3**

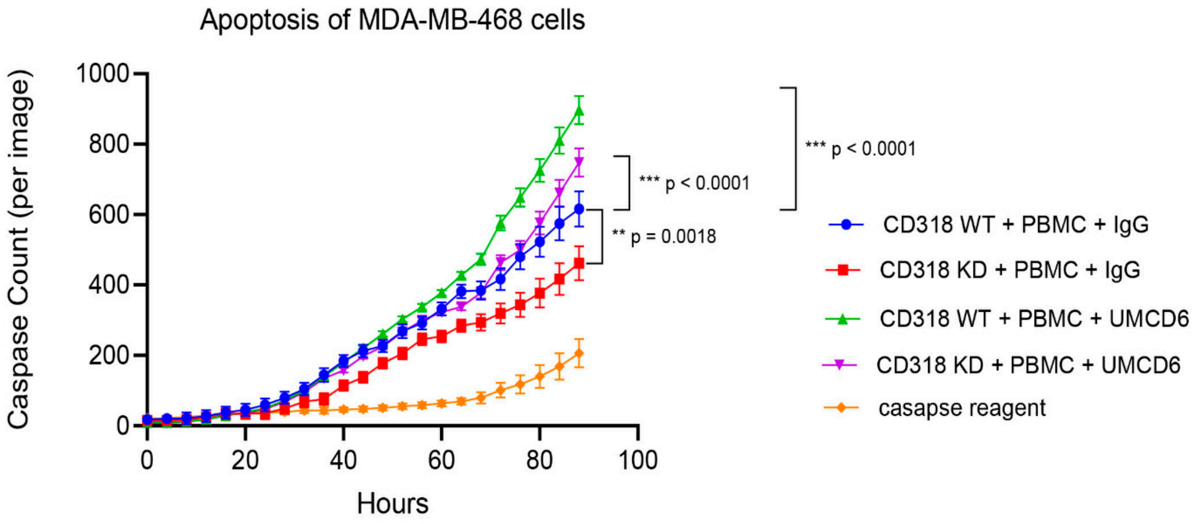


**Figure S3. CD318 knockdown reduces UMCD6-enhanced PBMC-mediated apoptosis of MDA-MB-468 breast cancer cells.** Wild-type and CD318-knockdown MDA-MB-468 breast cancer cells were used as target cells in PBMC-mediated cytotoxicity assays. Human PBMCs were pretreated with UMCD6 or isotype control IgG and cocultured with target cells. Tumor cell apoptosis was monitored using IncuCyte live-cell imaging by quantifying nuclear caspase reporter fluorescence. UMCD6 enhanced PBMC-mediated apoptosis of wild-type CD318-expressing MDA-MB-468 cells, whereas this effect was reduced in CD318-knockdown cells. Data are presented as mean ± SEM from replicate wells in a representative experiment. Statistical significance was determined by two-way ANOVA with multiple-comparisons testing; $P < 0.001$.
